# Bioprinting and biomaterials for dental alveolar tissue regeneration

**DOI:** 10.3389/fbioe.2023.991821

**Published:** 2023-04-14

**Authors:** Serge Ostrovidov, Murugan Ramalingam, Hojae Bae, Gorka Orive, Toshinori Fujie, Xuetao Shi, Hirokazu Kaji

**Affiliations:** ^1^ Department of Biomechanics, Institute of Biomaterials and Bioengineering (IBB), Tokyo Medical and Dental University (TMDU), Tokyo, Japan; ^2^ Institute of Tissue Regeneration Engineering, Dankook University, Cheonan, Republic of Korea; ^3^ Department of Nanobiomedical Science, BK21 PLUS NBM Global Research Center for Regenerative Medicine, Dankook University, Cheonan, Republic of Korea; ^4^ School of Basic Medical Science, Chengdu University, Chengdu, China; ^5^ Mechanobiology Dental Medicine Research Center, Dankook University, Cheonan, Republic of Korea; ^6^ UCL Eastman-Korea Dental Medicine Innovation Centre, Dankook University, Cheonan, Republic of Korea; ^7^ Department of Metallurgical and Materials Engineering, Faculty of Engineering, Atilim University, Ankara, Türkiye; ^8^ Department of Stem Cell and Regenerative Biotechnology, KU Convergence Science and Technology Institute, Konkuk University, Hwayang-dong, Seoul, Republic of Korea; ^9^ NanoBioCel Group, Laboratory of Pharmaceutics, School of Pharmacy, University of the Basque Country UPV/EHU, Vitoria-Gasteiz, Spain; ^10^ Bioaraba, NanoBioCel Research Group, Vitoria-Gasteiz, Spain; ^11^ Biomedical Research Networking Centre in Bioengineering, Biomaterials and Nanomedicine (CIBER-BBN), Vitoria-Gasteiz, Spain; ^12^ School of Life Science and Technology, Tokyo Institute of Technology, Yokohama, Japan; ^13^ Living System Materialogy (LiSM) Reseach Group, International Research Frontiers Initiative (IRFI), Tokyo Institute of Technology, Yokohama, Japan; ^14^ National Engineering Research Center for Tissue Restoration and Reconstruction, South China University of Technology, Guangzhou, Guangdong, China

**Keywords:** bioprinting, biomaterials, bioink, dental tissue engineering, dentistry

## Abstract

Three dimensional (3D) bioprinting is a powerful tool, that was recently applied to tissue engineering. This technique allows the precise deposition of cells encapsulated in supportive bioinks to fabricate complex scaffolds, which are used to repair targeted tissues. Here, we review the recent developments in the application of 3D bioprinting to dental tissue engineering. These tissues, including teeth, periodontal ligament, alveolar bones, and dental pulp, present cell types and mechanical properties with great heterogeneity, which is challenging to reproduce *in vitro*. After highlighting the different bioprinting methods used in regenerative dentistry, we reviewed the great variety of bioink formulations and their effects on cells, which have been established to support the development of these tissues. We discussed the different advances achieved in the fabrication of each dental tissue to provide an overview of the current state of the methods. We conclude with the remaining challenges and future needs.

## 1 Introduction

**FIGURE 1:**
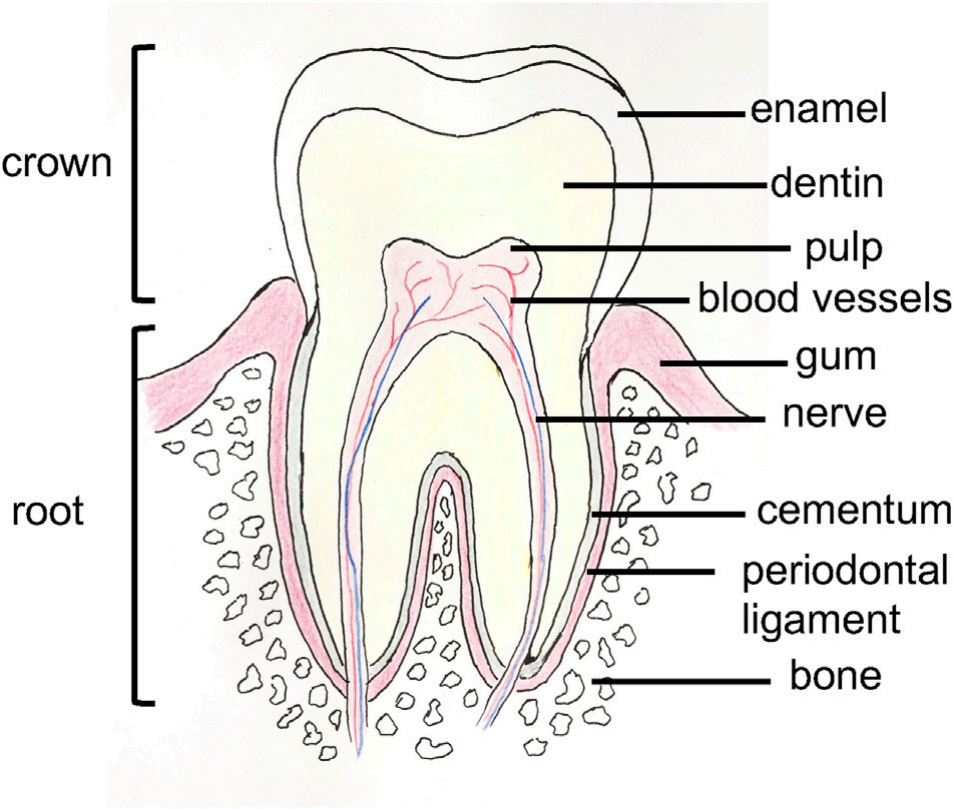
Anatomy of a tooth.

Oral health is an important part of general health and is a daily consideration for most people. Dental alveolar tissues are various (e.g., alveolar bone, dental pulp, teeth, periodontal ligament, gums, blood vessels, and nerves) and work synergistically to ensure daily physiological mastication, and digestive function (Ma et al., 2019). The tissues are organized in an ordered and complex spatial structure (Figure 1), with the involvement of different cell types, and exhibit different mechanical properties; as a result, tissues range from soft to hard (Goudouri et al., 2017). They may be subjected to different damages, including cavities, tooth loss, periodontitis, gingivitis, and bone defects. Achieving full restoration of teeth is very challenging. However, several strategies have been established to restore these tissues. Thus, in the case of caries, the infected tissue is removed, and the space is cleaned and filled with a synthetic inert material (Duarte Campos et al., 2020). However, if the caries is too significant, a root canal treatment will be performed, the dental pulp tissue will be sacrificed, and the tooth will lose important biological functions (e.g., sensory function, dentin production, immunology response) (Athirasala et al., 2017). Another well-known dental care is the use of dental implants to replace a lost tooth, and nearly 5 million dental implants are placed each year in the United States (Morrison and Tomlinson, 2021). In parallel with these current treatments, more regenerative strategies are being developed with the aim of restoring, maintaining, and replacing these dental alveolar tissues and functions. For example, advances have been made in bone tissue engineering and in dental pulp regeneration with the use of scaffolds and natural and synthetic hydrogels (Kim et al., 2009; Chen et al., 2010; Abbass et al., 2020). However, due to their variety, dental alveolar tissues create challenges for bioengineers, especially due to their strong interrelations; thus, these tissues should be considered more as one hybrid organ than separated tissues (Amoli et al., 2022). Furthermore, due to the variety of tissues, different biomaterials are needed with different mechanical properties to mimic the microenvironment of these tissues (Han et al., 2019). In recent years, 3D bioprinting has been considered a potential tissue engineering strategy to address this complexity. Indeed, 3D bioprinting allows the precise positioning of cells and matrix as the use of different cell types and materials in bioinks, and the speed of fabrication, resolution, and automation are advantageous (Vijayavenkataraman et al., 2018). In addition, 3D bioprinting has already been applied to the regeneration of many different tissues (Sun et al., 2020; Wang C.-Y.et al., 2021; Jain et al., 2022) such as skin (Weng et al., 2021), bones (Cheng et al., 2021), nerves (Cadena et al., 2021), heart (Wang Z. et al., 2021a), and skeletal muscle (Ostrovidov et al., 2019; Samandari et al., 2022). With images and data from computed tomography (CT) or magnetic resonance imaging (MRI), 3D bioprinting can produce patient personalized sophisticated constructs for the regeneration of dental alveolar tissue with complex architecture (Nesic et al., 2020a). Moreover, in combination with stem cell technology, which has provided an opportunity for the fabrication of human tissues, 3D bioprinting applied to dental and periodontal tissues is a very dynamic field of research (Mosaddad et al., 2022). Several advances have been achieved in 3D printed scaffolds, on which cells were seeded and in 3D bioprinted cell-encapsulated constructs for dentistry regeneration. Therefore, it is worth reviewing the latest advances in the field.

In this review, we begin by highlighting the different bioprinting techniques. Then, we review the different bioinks that have been fabricated and their use with different dental alveolar tissues (dental pulp, dentin, periodontal ligament, bone, gingiva, and whole tooth regeneration), providing an overview of the current state of the art. We then conclude by discussing the challenges and future developments of the field.

## 2 Bioprinting methods

**FIGURE 2:**
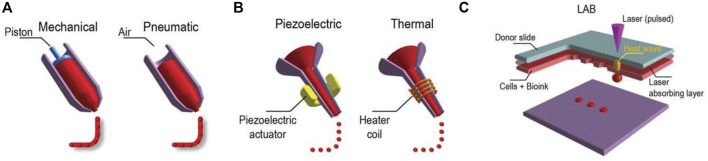
Three major bioprinting techniques **(A)** extrusion-based printers use mechanical or pneumatic dispensing systems to extrude the bioink, **(B)** to force bioink droplets out of the nozzle, inkjet printers use either a pulsed heater to heat the print head producing air bubbles or a piezoelectric actuator to generate localized pressure *via* ultrasonic waves, and **(C)** laser-assisted bioprinters (LABs) use a laser beam on an absorbing substrate to generate heat waves that dispense the bioink onto a substrate. Reprinted with permission from (Ostrovidov et al., 2019) ^©^ 2019 WILEY-VCH Verlag GmbH and Co. KGaA, Weinheim.

In additive manufacturing or 3D printing, 3D structures are fabricated *via* a computer and a computer-aided design (CAD). When the materials printed contain cells, the method is called 3D bioprinting, and the usual techniques used are inkjet bioprinting, extrusion-based bioprinting, laser-assisted bioprinting, and stereolithography (Figure 2). Readers interested in bioprinting methods can reference previous reviews (Ashammakhi et al., 2018; Ostrovidov et al., 2019) and a briefly summary of the topic is provided in the following. In inkjet printing, the deposition of bioink is performed drop by drop (Ma et al., 2019). To generate these droplets of bioink, different systems are used, such as acoustic, piezoelectric, hydrodynamic, electrostatic, thermic process, and microvalves. The printer head is synchronized with a motorized stage, and the 3D structures are fabricated layer-by-layer by raster scanning. This technique usually requires low viscosity bioinks (3.5–12 mPa/s) and is fast, and the cell viability after printing is high (>85%) (Mandrycky et al., 2016; Amoli et al., 2022). In extrusion-based printing the bioink is compressed into a nozzle by mechanical or pneumatic force. Through this technique, bioinks can be used with a wide range of viscosities (30 mPa/s-60 × 10^7^ mPa/s) (Mandrycky et al., 2016; Guzzi and Tibbitt, 2019). The 3D structures are fabricated by the deposition of lines or small beads of bioink and by raster scanning the printer head over the stage. The printer head then moves in the *Z* direction, allowing layer-by-layer fabrication. The technique is fast, and the cell viability after printing remains high (∼80%). In laser-assisted printing, a bioink with a viscosity of 1–300 mPa/s is loaded on a ribbon coated by a thin metallic film (gold or titanium) (Zennifer et al., 2022). A laser pulse induces metal vaporization, which ejects a droplet of bioink toward the substrate. This is a fast printing technique with high precision deposition, and the cell viability after printing is high (>95%). In stereolithography, a photosensitive resin is cured point by point by a laser beam to fabricate a 3D structure. The technique is fast, and the cell viability after printing is high (>85%) (Grigoryan et al., 2021; Khorsandi et al., 2021).

## 3 Bioinks

The bioink encapsulates the cells and therefore must mimic the extracellular matrix (ECM) of the targeted tissue, supporting cell proliferation and differentiation (Ostrovidov et al., 2019). It must also be printable and maintain a given shape after printing; therefore, its rheological properties and gelling time are important (Chung et al., 2013). Furthermore, a homogeneous distribution of cells should occur within the bioink without cell sedimentation to allow long-term bioprinting (Ferris et al., 2013). In addition, the bioink should be biodegradable, ideally at a rate that fits the growth of the biological tissue, to be naturally replaced by the ECM components secreted by cells. Hydrogels are 3D polymeric networks with high water content that are stable in water due to the crosslinking of their polymeric chains and exhibit viscoelastic properties that mimic natural ECM (Peters et al., 2021). Due to these characteristics, hydrogels are optimal materials for tissue engineering applications, and for 3D bioprinting (Rana et al., 2014). Hydrogels can be classified as natural, synthetic, and composites. Natural hydrogels used for tooth regeneration include collagen, gelatin, fibrin, hyaluronic acid (HyA), alginate, agarose, and the important seminatural hydrogel gelatin methacryloyl (GelMA) (Fatimi et al., 2022). In this natural group, we can add natural decellularized matrices (dECM), which are very efficient in supporting cells and signaling to cells (Chae and Cho, 2022). Synthetic hydrogels include polycaprolactone (PCL), poly (ethylene glycol) (PEG), poly (ethylene glycol) dimethacrylate (PEGDA), poly (lactic acid) (PLA), and poly (lactide-co-glycolide) acid (PLGA) (Madduma-Bandarage and Madihally, 2021). In addition, different materials, such as ceramics, minerals (e.g., hydroxyapatite (HA), β-tricalcium phosphate (β-TCP), silicates), carbon nanotubes, and metals (e.g., titanium, magnesium alloys), have also been used in dental applications (Ma et al., 2019; Morrison and Tomlinson, 2021; Amoli et al., 2022). Natural hydrogels have arginine-glycine-aspartic acid (RGD) motifs that favor cell attachment and motifs for matrix metalloproteinase that favor polymer biodegradation (Xie et al., 2019). They usually exhibit cell supportive properties but show weak mechanical properties (Cui et al., 2017). In contrast, synthetic hydrogels lack RGD motifs, but their mechanical properties can be finely tuned (Cui et al., 2017). The formulation of a new bioink should balance cell supportive properties, printability, and mechanical properties that try to fit the microenvironment of the targeted tissue (Fatimi et al., 2022). To this end, different materials are often combined in composition and concentration, offering a large spectrum of possibilities.

To properly regenerate a dental tissue, a cell type should be chosen and used with the bioink. Different cell types have been used in dental alveolar tissue regeneration. Mesenchymal stem cells (MSCs), one of the stem cell used, can be obtained from adult tissues and have been isolated from various tissues and fluids (e.g., adipose tissue, placenta, bone marrow, amniotic fluid, umbilical cord, and urine) (Hass et al., 2011; Cai et al., 2013; Ostrovidov et al., 2015; Zhou et al., 2021). Interestingly, until now, eight dental-derived mesenchymal stem cells have been identified with dental pulp stem cells (DPSCs), stem cells from human exfoliated deciduous teeth (SHED), periodontal ligament stem cells (PDLSCs), dental follicle progenitor cells (DFPCs), alveolar bone marrow stromal cells (ABMSCs), stem cells from the apical papilla (SCAP), tooth germ progenitor cells (TGPCs), and gingival-derived mesenchymal stem cells (GMSCs) (Stefańska et al., 2020). Other stem cell sources for dental applications are adipose-derived stem cells (ASCs) and induced pluripotent stem cells (iPSCs) (Radwan et al., 2020). In the following section, we describe different bioinks that have been used with different dental alveolar tissues.

### 3.1 Bioinks for dental pulp regeneration

Dental pulp is the soft connective tissue inside the tooth that is composed of collagen fibers, proteoglycans, and cells (e.g., fibroblasts, hDSPCs, and immune cells). Dental pulp exhibits viscoelastic properties, and its Young’s modulus is approximately 0.8 kPa. Odontoblasts, which are differentiated hDPSCs that produce dentin, are present at the interface with dentin. Dental pulp also contains a cell-free zone (zone de Weil) that houses a capillary network and nerves. This rich environment must be considered when designing a bioink for the regeneration of dental pulp to spatially control the localized differentiation of hDPSCs (Han et al., 2019).

Rosa et al. wrote a review on the different methods used to regenerate dental pulp (Rosa et al., 2022). Several studies have compared bioprinted constructs with their equivalent hydrogels. Thus, Yu et al. compared alginate-GelMA hydrogel scaffolds and alginate-GelMA bioprinted constructs with a Bioplotter EnvisionTec (extrusion bioprinting) toward hDPSC proliferation and differentiation. The results showed higher cell adhesion and proliferation on bioprinted constructs than on hydrogel scaffolds. Furthermore, after 14 days in mineralization medium, alizarin red S and alkaline phosphatase (ALP) staining showed that more calcium nodules and bonelike nodules were present in the bioprinted constructs than in the hydrogel scaffolds (Yu et al., 2019). In another study, Choi et al. used the mineral trioxide aggregate (MTA) to fabricate a GelMA-MTA bioink. MTA is a calcium silicate (CS)-based cement that contains tricalcium silicate, tricalcium aluminate, tetracalcium aluminoferrite, gypsum, bismuth oxide, and other mineral oxides (Santos et al., 2021). The researchers printed 3D scaffolds of GelMA and GelMA-MTA with a ROKIT Healthcare INVIVO 3D bioprinter (extrusion bioprinting) and seeded primary hDPSCs on them to evaluate their proliferation and differentiation. After 7 days of culture in differentiation medium, they observed the promotion of odontogenic differentiation through ALP measurement and expression of the genes dentin sialophosphoprotein (DSPP) and dentin matrix acidic phosphoprotein 1 (DMP-1) on GelMA-MTA bioprinted constructs (Choi et al., 2022). In the dental pulp complex, hDPSCs are present in the center of the teeth, while dentin is in the outer region. Therefore, to examine the possibility of spatial regulation of hDPSC differentiation, Han et al. used fibrin-based bioinks (fibrinogen/gelatin/hyaluronic acid/glycerol) with different concentrations of fibrinogen (5, 10, 15, 20 mg/mL) to spatially control the stiffness; thus, hDPSCs were bioprinted with the ITOP system (custom bioprinter, extrusion bioprinting) following a dental shape recorded by computed tomography (CT). After 15 days of culture in differentiation medium, the results showed spatial odontogenic differentiation with the central pulp region remained undifferentiated, whereas there was localized deposition of dentin in the outer region (Han et al., 2019). Moreover, to regenerate dental pulp tissue, Duarte Campos et al. evaluated cocultures of hDPSCs and primary human umbilical vein endothelial cells (HUVECs) encapsulated in different bioinks (0.2% collagen type I-0.5% agarose, 0.5% fibrin, and 0.3% collagen type I) deposited into root canals of bovine teeth *via* a hand-held bioprinter (DropGun Black Drop Biodrucker GmbH, *in situ* inkjet bioprinting). After 14 days of culture, new capillary networks were observed in the all tested samples (Duarte Campos et al., 2020). Similarly, Khayat et al. encapsulated hDPSCs and HUVECs in GelMA hydrogel and filled it into root segments (H 6 mm × D 3 mm) closed on one side with white mineral trioxide aggregate (WTMA). After 13 days of culture *in vitro* in osteogenic medium, the researchers implanted them subcutaneously in nude rats for 4 and 8 weeks. The results showed cellularized pulp-like tissue at day 13 of culture, whereas neovascularization was observed at 4 and 8 weeks postsurgery. Furthermore, cellular extension into dentin tubules and formation of dentin matrix were promoted (Khayat et al., 2016). Moreover, Athirasala et al. encapsulated odontoblasts (OD21) into a GelMA 15% hydrogel with a central engineered channel obtained by the sacrificial template technique. Then, they filled the channel with endothelial colony forming cells (ECFCs) and placed the construct into human teeth roots (H 9 mm x D 1.5 mm). After 7 days of culture in DMEM-EGM-2MV (1:1) medium, ECFCs formed a monolayer, and angiogenic sprouting was observed through the whole GelMA hydrogel with pulp-like tissue (Athirasala et al., 2017).

### 3.2 Bioinks for dentin regeneration

Dentin is a mineralized tubular structure that surrounds the dental pulp and is composed of hydroxyapatite (HA) and an organic matrix (35% by weight) of collagenous and noncollagenous proteins (e.g., DSPP, DMP-1, osteopontin (OP), osteocalcin (OCN)) (Zhang et al., 2014). Dentin has a Young’s modulus that ranges from 17–42 GPa following its mineral content. When designing a bioink for dentin regeneration the Young’s modulus and a certain microporosity (∼300 μm) are important parameters to consider.

**FIGURE 3:**
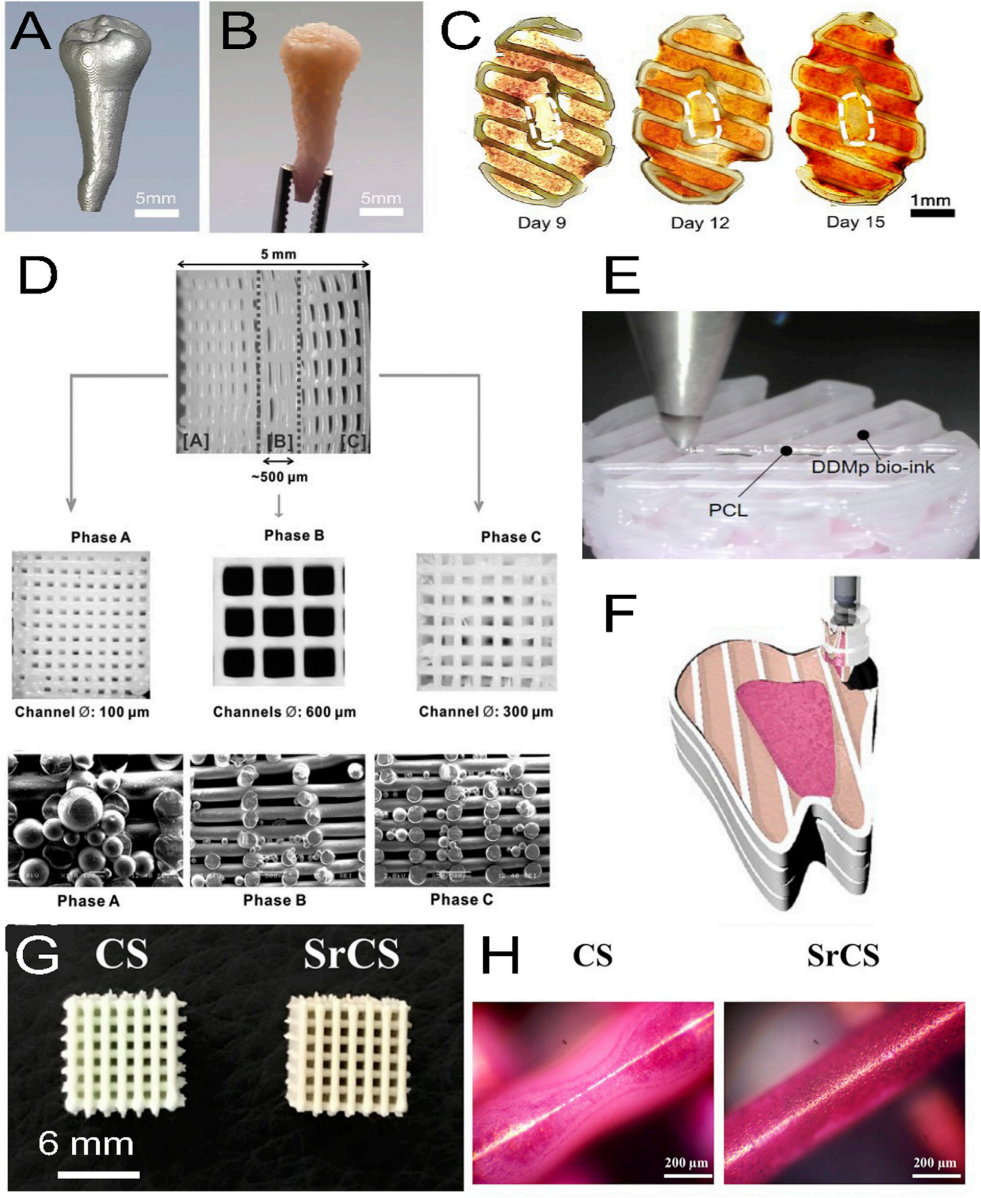
**(A)** Micro-CT image of a patient tooth, **(B)** with its 3D printed model with a fibrinogen-gelatin-hyaluronic acid-glycerol bioink loaded with hDPSCs. **(C)** Cross-sectional views of the construct stained with alizarin red S at different days of culture. The staining shows a spatial deposition of calcium, which corresponds to a spatial differentiation of hDPSCs that mimics the dental pulp complex, with an unmineralized central area (white dashed circles) similar to a pulp center and a mineralized outer area similar to a dentin region. **(D)** 3D printed triphasic scaffold of PCL-hydroxyapatite (HA) bioink used for PDL regeneration. Phase **(A)** A scaffold area with 100 μm microchannels and PLGA microspheres loaded with amelogenin was seeded with hDPSCs for dentin-cementum formation. Phase **(B)** A scaffold area with 600 μm microchannels and PLGA microspheres loaded with CTGF was seeded with hPDLSCs for periodontal ligament formation. Phase **(C)** A scaffold area with 300 μm microchannels and PLGA microspheres loaded with BMP-2 was seeded with hABSCs for alveolar bone formation. **(E)** Multimaterial bioprinting of a construct with a PCL bioink and a fibrinogen/gelatin/hyaluronic acid/glycerol/human demineralized dentin matrix (DDM 10%) bioink with hDPSCs for odontogenic differentiation. **(F)** Schematic showing the bioprinting strategy for the generation of dental pulp (fuchsia pink area) and dentin (light pink area) complexes, which were generated by depositing two different bioinks with hDPSCs in a printed PCL frame (white area). The whole construct was obtained by a layer-by-layer process. **(G)** Top view of 3D printed calcium silicate (CS) and strontium calcium silicate (SrCS) scaffolds used for bone regeneration **(H)** with alizarin red S staining at day 7 of culture. Sr promotes MSC osteogenic differentiation with increased calcium deposition. **(A–F)** adapted with permission from (Han et al., 2019)^©^2019 The Authors (open access), **(D)** Reprinted with permission from (Lee et al., 2014)©Mary Ann Liebert, Inc, **(G, H)** adapted with permission from (Chiu et al., 2019) (open access).

Different bioinks have been fabricated for dentin regeneration. For example, Mousavi Nejad et al. fabricated two scaffolds, PCL/45S5 Bioglass (BG) composite and PCL/HyA, by 3D printing with a 3DPL Bioprinter N2 (extrusion bioprinting) and evaluated them with hDPSCs for dentin and dental pulp regeneration. Both scaffolds allowed cell adhesion, and after 21 days of culture in differentiation medium, significantly higher expression of DSPP, OCN and DMP-1 was observed in the PCL/BG group due to the presence of bioglass, as demonstrated by gene analysis. It was concluded that PCL/BG was a favorable scaffold for dentin regeneration, whereas PCL/HyA was a favorable scaffold for dental pulp regeneration (Mousavi Nejad et al., 2021). In another study, Wu et al. fabricated a bioink of PCL/MTA for the regeneration of dentin and used an E-jetting custom system to fabricate the scaffold. However, the construct did not mimic natural dentin in composition and structure (no collagen, pore size 200 μm which is 100 times larger than natural dentin pore size) (Wu et al., 2016). Naseri et al. used a CELLINK bioprinter Biox (extrusion bioprinting) at 10–12 mm/s and 40–50 kPa with a nozzle of 518 μm diameter to bioprint a scaffold of collagen type I-hydroxyapatite-alginate that exhibits homogeneous repartition of pores with 2–4 μm sizes after being freeze-dried; this scaffold compositionally and microstructurally mimics natural human dentin. An occlusion test was performed by using polystyrene microparticles (2–3 μm diameter), and hMSCs were seeded on the scaffold to show cytocompatibility (Naseri et al., 2021). Moreover, decellularized matrices are very efficient in tissue engineering due to the signaling proteins they contain. Demineralized dentin matrix (DDM) has similar components than dentin but in a different organic/inorganic ratio. Several growth factors, such as transforming growth factors β1 (TGF-β1), BMPs, vascular endothelial growth factor (VEGF), fibroblast growth factor-2 (FGF-2), platelet derived growth factor (PDGF), and IGF-1, are also present in DDM, which makes DDM a very interesting biomaterial for dentin regeneration (Gao et al., 2019). Thus, Han et al. fabricated a fibrinogen/gelatin/HyA/glycerol/DDM particle bioink and evaluated its printability and its activity on hDPSC differentiation (Figure 3). They observed that the viscosity of the bioink increases with the concentration of DDM particles (1, 3, 5, 10% w/v). They obtained a minimal printed line width of 363 μm with fibrinogen/gelatin/HyA/glycerol bioink (without DDM particles) used as a control and 252 μm with the fibrinogen/gelatin/HyA-glycerol/DDM (10% w/v) bioink. Encapsulated hDPSCs (3×10^6^ cells/mL) were bioprinted with the ITOP system (custom bioprinter, extrusion bioprinting), and the constructs were placed in odontogenic medium for 15 days. The results showed high cell viability (>95%) at day 7 of culture, and alizarin red S staining at day 15 of culture showed that mineralization increased with increasing DDM particle concentration. Furthermore, the expression levels of DSPP and DMP-1 were 22.86- and 59.76- fold higher than those in the control group, respectively (Han et al., 2021). Furthermore, dentin and dental pulp both protect and nourish the whole tooth and interact together. To prevent dental pulp exposure during cavity treatment, dental pulp capping materials, such as calcium hydroxide (CH), are used. However, CH involves some drawbacks such as inducing dental pulp inflammation; therefore, the search for new dental pulp capping materials is ongoing. Decellularized matrix-like treated dentin matrix (TDM) powder has been used as capping material and has been shown to stimulate stem cells in dental pulp to secrete dentin and correctly regenerate the dentin structure (Li et al., 2011; Zhang et al., 2011; Guo et al., 2012; Yang et al., 2012). Thus, Chen et al. fabricated a TDM paste (TDMP), which is easier to customize in dental pulp capping treatment due to its plasticity, and evaluated its effect on the differentiation of hDPSCs. At 6 and 10 weeks post capping in miniature swine, the results showed that the thickness of the regenerated dentin was 371.94 μm and 541.41 μm, respectively, in the TMDP group *versus* 285.15 μm and 324.48 μm, respectively, in the CH group (Chen et al., 2017). Furthermore, Athirasala et al. fabricated a new bioink based on alginate and acid soluble dentin matrix extract (10, and 100 μg/mL). After optimizing the bioink, the researchers bioprinted SCAPs in alginate-dentin matrix bioink using a modified Hyrel 3D printer (extrusion printing) and placed the construct in odontogenic medium. They observed a high cell viability (>90%) after printing and an enhancement in ALP and RUNX2 gene expression at day 10 of culture (Athirasala et al., 2018).

### 3.3 Bioinks for periodontal ligament regeneration

Periodontal ligament (PDL) is a connective tissue that is mainly composed of collagen type I fibers and proteoglycans; PDL surrounds teeth roots and the interface between teeth and alveolar bones. The Young’s modulus of PDL is 5 × 10^6^ N/m^2^. It is a specialized tissue that embeds blood vessels, nerves, and several cell types (e.g., fibroblasts, osteoblasts, osteoclasts, PDLSCs), which show viscoelastic properties, contain proprioceptive sensors, anchor teeth and are involved in tissue regeneration and homeostasis of alveolar bones. The cementum interfaces the PDL and the dentin of the roots and is a hard and thin avascular mineralized tissue composed of 45%–50% HA, 50% organic matrix (mainly collagen type I, and collagen types III and XII, proteoglycans, osteopontin, osteonectin, osteocalcin, and sialoporotein), and cells (cementoblasts, PDLSCs, cementocytes) (Goudouri et al., 2017). Moreover, gingiva is the oral mucosa that covers alveolar bones. It is a highly vascularized tissue composed of a stratified epithelium (keratinized layer, granular layer, spinous layer, and basal layer) and a fibrous connective tissue named the lamina propria. Gingiva thickness ranges from 0.3–6.7 mm, and its Young’s modulus ranges from 1–5 MPa. Gingiva contains several cell types (mainly keratinocytes, Langerhan’s cells, and Merkel cells) (Chen et al., 2015). Gingiva is a constituent of the periodontium.

Park et al. wrote an interesting review on periodontal complex regeneration. The periodontal complex consists of tooth supportive tissues and includes the cementum, the PDL, the gingiva, and the alveolar bone. They presented several advances techniques, such as scaffolding, cell sheet technology, and 3D printing, used in periodontal regeneration (Park et al., 2016). Thus, Dan et al. fabricated a calcium phosphate (CaP)-coated PCL for the transplantation of cell sheets to denuded root surfaces of an athymic rat periodontal defect model. Human periodontal ligament cells were cultured on thermosensitive poly (N-isoproplyacrylamide) (PiPAam) dishes. Several cell sheets (3 layers) were then harvested on CaP-PCL and transplanted onto the defect. Four weeks after transplantation, the results showed that CaP-PCL alone promoted bone formation, whereas cell sheets induced the formation of PDL (Dan et al., 2014). Furthermore, Reis et al. fabricated a PLGA/CaP bilayer scaffold with flat-smooth outer layer and a 1 mm thick rough macroporous inner layer, which was implanted in a dog model of a furcation defect. The results showed the formation of new cementum, bone, and periodontal ligament with Sharpey fiber insertions (Carlo Reis et al., 2011). Moreover, Lee et al. used 3D printed with a 3D-Bioplotter EnvisionTec (extrusion bioprinting) to 3D print a multiphasic scaffold of PCL-HA (90:10 wt%) with interlaid strands (diameter 100 μm) which defined interconnected microchannels with diameters of 100, 600, and 300 μm in phases A, B, and C, respectively. In addition, PLGA microspheres encapsulating recombinant human amelogenin, connective tissue growth factor (CTGF), and BMP-2 were tethered to phases A, B, and C, respectively. The results after 4 weeks of culture in defined medium or implanted *in vivo* subcutaneously in immunodeficient mice showed that hDPSCs seeded on this scaffold differentiated into dentin/cementum, PDL, and alveolar bone (Lee et al., 2014). Periodontal regeneration implies the restoration of multiple tissue types, spatial fibrous tissue organization, and functional restoration. To overcome this structural complexity, Park et al. 3D printed a wax mold with a Solidscape printer Model Maker II (extrusion printing) to cast biomimetic hybrid scaffolds with specific polymer areas to engineer bone in the PCL compartment and PDL in the PGA compartment. Primary human gingival fibroblasts (hGF) and hPDLCs in fibrinogen solution were seeded in the PCL and PDL compartments, respectively. After achieving gelation with thrombin, human dentin slices with exposed dentinal tubules were used to cap the constructs. The whole constructs were then transplanted subcutaneously into the dorsa of an immunodeficient mouse model for 6 weeks. The results showed that ligament and bone tissues were generated with specific localization and organization (Park et al., 2010). In another study, Rasperini et al. reported on a customized scaffold clinically transplanted in a periodontal defect. Patient with periodontal defect underwent a CT scan and a biodegradable PCL scaffold containing 4% HA was 3D printed with a selective laser sintering EOS Formiga P100 System, sterilized by ethylene oxide, immersed in recombinant human PDGF (0.3 mg/mL) for 15 min, and transplanted on the defect. The scaffold remained in place for 1 year without signs of inflammation or dehiscence. However, at 13 months postoperation the site showed dehiscence and wound failure, and the scaffold was removed and analyzed. The researchers concluded that the choice of PCL biomaterial was not ideal and that another biomaterial with a highly porous structure and a faster resorption time would be better (Rasperini et al., 2015). After optimizing the conditions of printing, Raveendran et al. bioprinted primary hPDLCs with a Gesim Bioscaffolder 3.1 (inkjet printing) in 12.5% GelMA at 11 mm/s, 135 kPa, and a 25 G needle. The cells proliferated well in the construct and colonized it within 2 weeks (Raveendran et al., 2019).

GMSCs have been differentiated into neural cells, endothelial cells, osteoblasts, adipocytes, chondrocytes, and myocytes and are often used for bone and neural regeneration (Grawish, 2018). Sun et al. have injected GMSCs into the tail vein of mice with periodontitis. They observed that GMSCs could target the periodontal injury site and promote periodontal ligament and alveolar bone tissue regeneration (Sun et al., 2019). Although many publications have used GMSCs, within the field of bone and neural regeneration, there are usually less discussion on gingiva regeneration, especially regeneration through bioprinting. An analogy is sometimes made between skin regeneration and gingival regeneration. Nesic et al. wrote an interesting review on 3D bioprinting for oral soft tissues (Nesic et al., 2020b). In a recent study, Yi et al. fabricated a bioink of injectable platelet-rich fibrin (iPRF)-alginate-gelatin. Six milliliters of human whole blood without anticoagulant was centrifuged at 700 rpm at room temperature (RT). The plasma liquid was harvested and mixed at 10%, 30%, and 50% volume ratios with 4% sodium alginate-8% gelatin-α-MEM culture medium bioink. Primary hGFs (1×10^6^ cells/mL) were loaded in the bioink and bioprinted with a Medprin bioprinter 2.0 (extrusion bioprinting) at 10 °C, 30 mm/s, and a nozzle diameter of 260 μm. The results showed a sustained release of growth factors over 2 weeks, excellent cell attachment, and promotion of hGF cell proliferation. At 4 weeks post-implantation in nude mice, a 20% increase in new blood vessels was observed in the scaffold (Yi et al., 2022).

### 3.4 Bioinks for alveolar bone regeneration

Alveolar bones are the parts of the mandible and maxilla that anchor the teeth in tooth sockets (Goudouri et al., 2017). They are mainly trabecular bone, composed of 65% mineralized tissue (calcium deficient HA), 30% organic matrix (mainly collagen type I and noncollagenous proteins such as osteopontin, osteonectin, sialoporotein), water 15%, and cells (∼5%) which are mainly osteoclasts, osteoblasts, and osteocytes. Their Young’s modulus ranges between 0.9 × 10^9^–13.7 × 10^9^ N/m^2^. Alveolar bones are covered by a membrane (periosteum). When designing a bioink for alveolar bone regeneration the load (while chewing), Young’s modulus, porosity, and bioactivity of different cell types must be considered.

Leyendecker Junior et al. performed an interesting analysis of the literature from 1984 to 2017 concerning the use of DPSCs and SHED for bone tissue engineering and wrote a review (Leyendecker Junior et al., 2018). Furthermore, Duarte Campos et al. used a custom-made inkjet bioprinter to fabricate agarose-collagen type I hydrogel constructs and studied the effect of their stiffness (low, medium, high) on MSC osteogenic differentiation (Table 1). The results showed that the viscosity of the hydrogels increased with the agarose concentration, whereas the high stiffness blended hydrogel was 3 times greater than that of pure agarose hydrogels. Furthermore, higher osteogenic differentiation was observed when cells were encapsulated in lower stiffness agarose-collagen hydrogel, which allowed the spreading and branching of cells (Duarte Campos et al., 2016). However, the compressive modulus of these hydrogels ranged from 18.1 kPa to 89.1 kPa, which is very low compared to the 110 MPa of bone. In another study, Park et al. evaluated the cell viability and differentiation of hDPSCs encapsulated in a BMP-2-conjugated GelMA bioink. They used a synthetic BMP-2 mimetic peptide (KIPKASSVPTELSAISTLYL) and checked the rheological property of this new bioink. They observed high cell viability (>90%) after bioprinting with the integrated organ printing system (ITOP system, extrusion bioprinting) and cell proliferation. After 4 weeks in osteogenic differentiation medium, calcium deposition was evaluated by alizarin red S staining and showed that 55% of the construct was calcified. Furthermore, high expression of DSPP and OCN genes was observed in the construct (Park et al., 2020). To increase mineralization, Kim et al. mixed collagen type I (5%), and bone-dECM (5%) with β-TCP (0, 10, 20, 30, 40% fraction weights) to obtain different bioinks; then, they encapsulated 1×10^7^ hDPSCs/mL into these bioinks that were bioprinted with a three-axis robot system DTR3-2210-T-SG (DASA Robot, extrusion printing). They selected the bioink containing 20% wt of β-TCP on the basis of high cell viability (>95%) and placed the bioprinted constructs in osteogenic differentiation medium. After 21 days in culture, they evaluated osteogenic (ALP, OCN, OPN) and odontogenic (DSPP, DMP-1) differentiation and observed significant enhancement in both differentiation (Kim et al., 2022). Similarly, Yang et al. fabricated a bioink of GelMA-methacrylated hyaluronic acid with or without collagen type I, in which they encapsulated osteocytes (IDG-SW3, 1×10^7^ cells/mL) that were printed with a 3D-Bioplotter EnvisionTec (extrusion bioprinting). After 28 days of culture in mineralization medium, the results showed high DMP-1 and calcium deposition, whereas cells exhibited a typical dendritic morphology with aligned and dense dendrites and were characterized as mature osteocytes by the high expression of SOST protein (Yang et al., 2020). In another study, Son et al. examined the differences between primary mouse alveolar bone-derived cells (mABDCs) and primary mouse long bone-derived cells (mLBDCs). They observed that both exhibit similar osteoblastic characteristics, morphology, and proliferation rates, but show distinct expression of epithelial-mesenchymal interaction (EMI)-related genes. Thus, among these genes, BMP-4 has a critical effect on bone formation in mABDCs but not in mLBDCs (Son et al., 2020). Moreover, Chiu et al. fabricated a strontium-containing calcium silicate (SrCS) scaffold by 3D printing with a Gesim Bioscaffolder 3.1 (inkjet printing) and evaluated it relative to a CS scaffold for osteoinduction. Analysis showed that the SrCS scaffold exhibits macropores of 0.5 mm and a compressive modulus two times higher than that of the CS scaffold. Moreover, when placed in simulated body fluid (SBF) solution, SrCS released more Ca, Si, Sr, and P ions, whereas both scaffolds induced apatite formation. When MSCs were seeded on the scaffolds, compared to CS, SrCS more greatly stimulated cell attachment, proliferation, and osteogenic differentiation with higher levels of ALP, BSP, OPG, and OC. When implanted *in vivo* in a rabbit model, 2 times more new bone formed in SrCS than in CS at 4 weeks postsurgery (Chiu et al., 2019). Similarly, Wang et al. bioprinted with Gesim Bioscaffolder 3.1 (inkjet printing) a bilayer scaffold of collagen-SrCS with hGFs encapsulated in collagen as the top layer and SrCS as the bottom layer. The bilayer scaffold supports cell viability and proliferation, as observed over an 8-week period. Furthermore, the bilayer scaffold promoted the secretion of FGF-2, BMP-2, and VEGF by hGFs. After the scaffold was implanted in rabbits for 12 weeks, new bone formed around and in the bilayer scaffold, while it only formed around the single-layer SrCS scaffold used for comparison, as determined by μCT analysis and Von Kossa staining (Wang Z. et al., 2021b).

**TABLE 1:** Overview of bioinks used for dental alveolar tissue regeneration with their effects.

Tissue	Matrix	Cell source	Construct type	Printer and printing type	Effects	References
Dental pulp	Alginate/GelMA	hDPSCs (Cells seeded)	-Printed constructs	-Bioplotter Envision Tec	Printed constructs > hydrogel scaffolds. Higher cell attachment, and proliferation. Higher mineralization (Calcium up/ALP up)	Yu et al. (2019)
	Alginate/GelMA		-Hydrogel scaffolds	-Extrusion printing		
Dental pulp	GelMA-MTA	hDPSCs (Cells seeded)	Printed construct	-Rokit Healthcare *IN VIVO* printer	GelMA-MTA increases mineralization ALP, DSPP, DMP-1 up	Choi et al. (2022)
	GelMA			-extrusion printing		
Dental pulp	fibrinogen/gelatin/hyaluronic acid/glycerol with [fibrinogen] 5–20 mg/mL	hDPSCs (Cells encapsulated)	Bioprinted constructs	-ITOP system	Spatial differentiation (undifferentiated in central pulp area and dentin deposition in outer area)	Han et al. (2018)
				-extrusion bioprinting		
Dental pulp	−0.2% Collagen type I/0.5% agarose	hDPSCs/HUVECs (Cells encapsulated)	Bioprinted constructs in bovine root canal (hand-held bioprinter)	-DropGun Black Drop Biodrucker	Vascularized dental pulp with the three bioinks. (Collagen/agarose bioink has better mechanical properties)	Duarte Campos et al. (2020)
	−0.5% fibrin −0.3% Collagen type I			-inkjet bioprinting		
Dentin	PCL/45S5 bioglass	hDPSCs (Cells seeded)	Printed constructs	-3DPL bioprinter N2	PCL/bioglass induced dentin formation DSPP, OCN, DMP-1 up	Mousavi Nejad et al. (2021)
	PCL/Hyaluronic acid			-extrusion printing	PCL/Hyaluronic acid induced dental pulp formation	
Dentin	Collagen type I/hydroxyapatite/alginate	hMSCs (Cells seeded)	Printed constructs	-CELLINK bioprinter Biox	Constructs mimic natural dentin. Pores size 2–4 μm. Good cell attachment and proliferation	Naseri et al. (2021)
				-extrusion printing		
Dentin	Fibrinogen/gelatin/10% DDM particles	hDPSCs (Cells encapsulated)	Bioprinted constructs	-ITOP system	Mineralization increases with increase [DDM] DSPP, DMP-1 up	Han et al. (2021)
				-extrusion bioprinting		
Dentin	Alginate/dentin matrix extract (10 and 100 mg/mL)	SCAPs (Cells encapsulated)	Bioprinted constructs	-Hyrel 3D printer	Optimized printing parameters. Good cell viability. ALP, RUNX2 up	Athirasala et al. (2018)
				-extrusion bioprinting		
Periodontal ligament	PCL/Hydroxyapatite (+ PLGA microspheres with amelogenin or CTGF or BMP2)	hDPSCs (Cells seeded)	Multi-phasic scaffolds (with A, B, C areas)	-Bioplotter Envision Tec	Spatial cell differentiation. Formation of dentin-cementum, PDL, and alveolar bone	Lee et al. (2014)
				-Extrusion printing		
Periodontal ligament	PCL/PGA	hGFs, hPDLSCs (Cells seeded in A, B areas, respectively)	Multi-phasic scaffolds (with A, B areas)	-SolidScape printer Model Maker II	Spatial cell differentiation. Formation of cementum, ligament (with oriented fibers), and bone	Park et al. (2010)
				-Extrusion printing		
Periodontal ligament	PCL/4% hydroxyapatite	No cells, but PDGF	Printed scaffold implanted in human	-Formiga P100	No inflammation, no problem for 1 year. At 13 months, dehiscence and wound failure. Scaffold was removed	Rasperini et al. (2015)
				-selective laser sintering		
Periodontal ligament	GelMA 12.5%	hPDLSCs (Cells encapsulated)	Bioprinted constructs	-Gesim Bioscaffolder 3.1	Optimized printing parameters. Good cell viability and proliferation	Raveendran et al. (2019)
				-inkjet bioprinting		
Gingiva	4% alginate/8% gelatin/(10, 30, 50%) platelet rich fibrin	hGFs	Bioprinted constructs	-Medprin bioprinter 2.0	Optimized printing parameters. Good cell viability and proliferation	Yi et al. (2022)
		(Cells encapsulated)	Implanted 4, 8 Wks in nude mice subcutaneously	-Extrusion bioprinting	Host cell infiltration Neovascularization	
Alveolar bone	Agarose/Collagen type I (with low, medium, high stiffness)	hMSCs (Cells encapsulated)	Bioprinted constructs	-custom-made bioprinter	Higher osteogenic differentiation in low stiffness bioink Alizarin red staining, ALP, COL1, RUNX2 up	Duarte Campos et al. (2016)
				-Inkjet bioprinting		
Alveolar bone	BMP2 peptide-conjugated GelMA	hDPSCs (Cells encapsulated)	Bioprinted constructs	-ITOP system	Good cell viability and proliferation. High calcifi-cation. DSPP, OCN up	Park et al. (2020)
				-extrusion bioprinting		
Alveolar bone	Collagen type I 5%/bone dECM 5%/β-TCP 20%	hDPSCs (Cells encapsulated)	Bioprinted constructs	-three axis DASA Robot system	Osteogenic and odontogenic differentiation. ALP, OCN, OPN up DSPP, DMP-1 up	Kim et al. (2022)
				-Extrusion bioprinting		
Alveolar bone	GelMA/HAMA	IDG-SW3 (Cells encapsulated)	Bioprinted constructs	-Bioplotter Envision Tec	After 28 days culture, high DMP-1 and calcium deposition. Cx43, Sost, Phex up, high dendrite density	Yang et al. (2020)
	GelMA/HAMA/Collagen type I			-Extrusion printing		
Alveolar bone	SrCS (strontium calcium silicate)	hMSCs (Cells seeded)	Printed constructs	-Gesim Bioscasffolder 3.1	Higher cell attachment, proliferation, differentiation with Sr. ALP, BSP, OPG, OC up	Chiu et al. (2019)
				-inkjet bioprinting		
Alveolar bone	Collagen/SrCS (bilayers)	hGFs (Cells encapsulated)	Bioprinted constructs Implanted 12 Wks in rabbit subcutaneously	-Gesim Bioscasffolder 3.1	Promotes FGF2, BMP2, VEGF secretion. Bone formation around and in the construct (bilayers), around only (single layer)	Chen-Ying Wang et al. (2021)
	SrCS (single layer)			-inkjet bioprinting		
Alveolar bone	Octapeptide/amorphous Mg phosphate	hDPSCs (Cells encapsulated)	Bioprinted constructs	-RegenHU printer	Promotes osteogenic differentiation. High mineralization. Increases bone formation and bone density	Dubey et al. (2020)
			Implanted 4, 8 Wks in rats calvarial defects	-inkjet bioprinting		

### 3.5 Bioinks for whole tooth engineering

An early attempt to regenerate a whole tooth was made by Kim et al., who printed a molar-shaped scaffold with a PCL (80%)/HA (20%) bioink (without cells) through 3D-Bioplotter EnvisionTec (extrusion bioprinting); in addition, they used a cell-homing strategy to condense multiple cell lineages into the scaffold for the generation of multiple tissues. They coated the scaffold with a blend of stromal derived factor-1 (SDF1, 100 ng/mL) and bone morphogenic protein 7 (BMP-7, 100 ng/mL) in a collagen type I solution and implanted the scaffold subcutaneously in rats for 9 weeks. The results showed new alveolar bone formation, mineralization, PDL formation, and angiogenesis (Kim et al., 2010). Interestingly, it was determined that the epithelial/mesenchymal cell interface observed in the early stage of tooth formation was essential, and it was shown that the regeneration of a whole tooth was possible. Although these studies did not involve a printing technique, we present the results due to their importance. However, since cell localization is important in the process, with new developments, a printing technique may be useful for whole tooth regeneration. Thus, Nakano et al. fabricated a tooth germ by encapsulating compartmentalized epithelium- and mesenchymal-derived stem cells at a high density (5×10^8^ cells/mL) in collagen hydrogel. The tooth germ generated the correct tooth structure in *vitro* cultures, as in *vivo* subcutaneous transplantation with penetration of blood vessels and nerves (Nakao et al., 2007; Ikeda et al., 2009). Similarly, Oshima et al. generated a tooth bud by compartmentalizing epithelium- and mesenchymal-derived stem cells. They placed this tooth bud in a plastic ring for size control and transplanted it into the mouse subrenal capsule for 2 months, which generated a tooth unit surrounded by alveolar bone. Then, the team investigated the potential engraftment of such a tooth unit into a defect in a mouse molar region of alveolar bone. Full bone integration was observed at 30 days post-surgery with regeneration of alveolar bone (Oshima et al., 2011). Oshima and Takuji wrote a review about the evolution of techniques used for whole tooth regeneration. They described the following methods: the scaffold method, the cell aggregation method, and the organ germ method (Oshima and Tsuji, 2014). In the scaffold method, epithelial and mesenchymal stem cells are seeded on biodegradable materials and the whole tooth is generated (Iwatsuki et al., 2006; Sumita et al., 2006; Yelick and Vacanti, 2006; Honda et al., 2007). The major drawbacks of this method are the irregularities in tooth production and in tissue structure full achievement. In the cell aggregation method, an aggregate of dental epithelial and mesenchymal cells is fabricated, and a tooth is generated by cell reorganization. However, the frequency of tooth formation and correct tissue formation are not secured (Yamamoto et al., 2003; Hu et al., 2006). In the organ germ method, epithelial and mesenchymal stem cells at high cell density are compartmentalized into a collagen gel. The tooth bud generates a whole tooth that is structurally correct *in vitro* and *in vivo* when transplanted into a jawbone (Oshima et al., 2011). Thus, Wen et al. fabricated a tooth germ by mixing hemisphere mouse iPS and mouse mesenchymal stem cells in a collagen type I. Then, they added epithelial cells to the adjacent area. The construct was placed in a 12-well insert culture plate in odontogenic induction medium for 5 days and then transplanted into the mouse subrenal capsule for 4 weeks. The results showed that the iPS alone group did not form tooth-like structures, epithelial cells with mesenchymal stem cells but without iPS cells formed irregular tooth-like structures, and only the 3 cell types together formed mature tooth-like structures similar to normal teeth (Wen et al., 2012). The organ germ method (or tooth bud model) has also been used by Smith et al., who encapsulated porcine dental epitaxial (pDE) progenitor cells, porcine dental mesenchymal (pDM) progenitor cells, and HUVECs in 3% and 5% GelMA hydrogels, which were cultured in osteogenic medium. After 3 and 6 weeks of culture *in vitro*, the results showed robust expression of DSPP, whereas the marker of bone differentiation was weak. Furthermore, after 6 weeks of subcutaneous implantation in rats, the explant analysis showed mineralized tissue formation, collagen type I and type III deposition, neovascularization, and bone formation (Smith et al., 2017). Furthermore, Zhang et al. used porcine decellularized tooth buds as a matrix, seeded them with porcine dental epithelial cells, human dental pulp cells, and HUVECs, and then implanted them in the mandible of mini-pigs for 3 and 6 months. The results showed successful whole tooth formation with enamel, pulp, dentin, periodontal ligament, and tooth roots. However, not all implants form mature teeth (Zhang et al., 2017).

## 4 Bioprinting regulations for dentistry

Since Charles Hull invented stereolithography in 1983, substantial progress and development have been achieved with 3D printing and bioprinting, and these technologies have been applied in various fields such as tissue engineering, regenerative medicine, personalized medicine, prostheses, implants, drug fabrication, and medical education (Ashammakhi et al., 2018; Ashammakhi et al., 2019; Cheng et al., 2021). Driven by the technological development of 3D bioprinters and biomaterials, numerous bioprinting companies have appeared on the market, among which 80% are established companies and 20% are start-ups (Santoni et al., 2022). In terms of business, the bioprinting market in the United States was evaluated in 2019 at $586.13 million and is expected to reach $1,949.94 million by 2025 (Santoni et al., 2022). As bioprinting is a new rising technology that is being dynamically developed there is also a strong need for regulation and standardization.

Different countries worldwide have established regulatory agencies to produce laws and regulations, which ensure the safety of new medicinal products and allow these products to enter clinical trials and the market. These agencies include the US Food and Drug Administration (FDA), the Central Drug Standards Control Organization (CDSCO) in India, the European Medicines Agency (EMA) in Europe, the Federal Service for Control over Healthcare and Social Development (Roszdravnadzor) in Russia, the Pharmaceutical and Medical Devices Agency (PMDA) in Japan, and the China Food and Drug Administration (CFDA) in China. 3D printed constructs, implants, and prostheses, without cellular components are usually regulated by these agencies as medical devices, whereas 3D bioprinted tissue engineered medical products (TEMPs) that contain cellular components are usually regulated as biologics or drugs (Sekar et al., 2021). For commercial purposes, 3D printed/bioprinted products should be fabricated under Good Manufacturing Practices (GMP) using clinical grade materials and be tested with Good Clinical Practice (GCP). The International Organization of Standards (ISO) and the American Society for Testing and Materials (ASTM) are the organizations internationally recognized for producing standardization guidelines for biomaterials and medical devices to ensure the product characteristics, their qualities, and the quality of the fabrication process and product analysis (Simon et al., 2014). These two organizations have technical committees and subcommittees that regularly establish new standards or revise previous standards (for example, ISO/ASTM 52900 and ISO 17296 series on 3D printing, ISO/TS 22911:2016 on dentistry and the preclinical evaluation of dental implants, ISO 7405:20,018 on the evaluation of the biocompatibility of medical devices used in dentistry, and ISO 22803 on membrane materials for guided tissue regeneration in oral and maxillofacial surgery).

For example, the FDA regulates 3D printed products for dentistry as medical devices based on their risks to health and classifies them into classes I, II, and III (Schuh and Funk, 2019). Class I includes devices presenting low risk for health, such as toothbrushes, surgical guides, and custom trays, and manufacturers must show that their product is biocompatible and produce the mechanical performance data (for FDA Products Classification see: https://www.accessdata.fda.gov/scripts/cdrh/cfdocs/cfPCD/PCDSimpleSearch.cfm). The ISO 10993 series focuses on “Biological evaluation of medical devices”, and these guidelines can be used for the preclinical evaluation of the biocompatibility of materials (Schuh and Funk, 2019). Class II includes devices that present moderate risk for health, such as dental implants, mouthguards, and aligners, and manufacturers must show that their product is biocompatible, that the contact between the device and the body does not induce complications and that the product is substantially equivalent to a product already on the market. When this equivalence is shown, a 510(k) clearance (premarket notification) is delivered (https://www.fda.gov/medical-devices/device-approvals-denials-and-clearances/510k-clearances). Class III includes devices with high risk for health, and the regulation is more severe, requiring higher safety assessment; usually 3D printed dental products fall into Class I and II. However, the product is sometimes not classified yet (e.g., plastic mouthguard fitted to the teeth, code OCO), and sometimes the product is exempted from the 510(k) clearance (e.g., additively manufactured preformed resin denture tooth, code PZY) despite being in Class II.

For the 3D bioprinted products with cellular components, the product combines a synthetic scaffold matrix and a cellular part qualifies in the United States as an HCT/Ps (human cell, tissues and cellular and tissues-based products, https://www.fda.gov/media/70689/download; and https://www.fda.gov/media/82724/download) as defined in the Code of Federal Regulation (21CFR Part 1271.3) based on the Public Health Service act (PHS Act). Since the cells have been mixed with the matrix (more than minimally manipulated), the product is then regulated as a biologic under Section 351 of the PHS Act, and a premarket notification, a new drug application (NDA), and a Biologics License Application (BLA) are necessary (Hourd et al., 2015; Ricles et al., 2018; Sekar et al., 2021).

Regulations between different countries may be similar but each country also has its own specificities; thus, clearance obtained in one country is not automatically recognized in another country. However, when a country does not have a regulatory agency, clearance might be first required from a country with an agency, before national regulation is considered. In Europe (EU), medical devices (no cellular component) are classified in Class I (low risk), Class IIa (low to medium risk), Class IIb (medium to high risk), and Class III (high risk), and products with cellular components are considered drugs or biologics. The regulation 2017/745 deals with medical devices, whereas the Directive 2004/23/EC covered transplants, tissues or cells of human origin, or their derivatives (https://op.europa.eu/en/publication-detail/-/publication/83bdc18f-315d-11e7-9412-01aa75ed71a1/language-en/format-PDF/source-58036705, https://eurlex.europa.eu/LexUriServ/LexUriServ.do?uri=OJ:L:2004:102:0048:0058:en:PDF).

## 5 Conclusion and perspective

Bioengineering is causing a major shift in the approach of dental treatments. Until now, traditional dental restoration techniques were the best possible with the technology available but were globally based on cleaning, inserting inert biomaterials for capping, protecting teeth and restoring a shape; thus, teeth would gradually lose parts of their functionalities and vitalities. In contrast, the aim of tissue engineering is to prevent the teeth from losing function and regenerate the damaged parts of tissue and functions. 3D bioprinting allows the precise deposition of cells, matrix, and signaling components to fabricate sophisticated constructs; these constructs show potential to revolutionize dental treatments and accelerate a rapid transition away from traditional restoration techniques toward bioengineered solutions. Currently, many efforts are being made to develop new bioinks that support cell proliferation and differentiation for dental alveolar tissue regeneration. Interestingly, papers have usually shown that bioprinted contructs are superior to hydrogel scaffolds (of the same material composition) in terms of cell support and cell differentiation. To provide additional guidance effects on cells and improve tissue regeneration, more complex bioinks may integrate signaling molecules, such as growth factors or peptides. For example, BMP-2 and BMP-7 have been approved by the FDA and may be used to induce tissue mineralization. The native protein may be replaced by a synthetic peptide, such as a haptamer, to reduce the cost, and its conjugation with the bioink leads to a long lasting effect rather than a short inducing effect and a faster clearance by proteases. Furthermore, due to the strong interrelations between dental alveolar tissues, multimaterial and multicellular bioprinting may often be needed. We have provided several examples of multiphasic constructs that have been fabricated, especially for periodontal complex regeneration, in which several tissues were generated simultaneously with proper orientation and integration. The developments of these bioprinting techniques should increase with the development of new multimaterial bioinks that support multicell types and compartmentalization. Moreover, stem cells are an inhexaustible source of cells for human organ regeneration that can differentiate into several tissue types. Different stem cells are available for dental regeneration, and among them, hDPSCs are frequently used, as shown by the literature. Another source of abundant and available stem cells could be adipose-derived stem cells (ASCs), which can differentiate into dental pulp, dentin, cementum, and periodontal ligament PDL. Due to the ability to reprogram adult cells into less differentiated cells, named induced pluripotent stem cells (iPSCs), ASCs are a very promising cell source of progenitors for dental regeneration. The technology to reprogram cells into iPSCs has progressed well; rather than using direct reprogramming with the insertion of transcription factors *via* lentivirus into cells, small molecules are now used in culture medium to induce specific signaling and to prevent viral components (Ostrovidov et al., 2023). However, the use of undifferentiated cells (e.g., MSCs, iPSCs) for transplantation has raised several concerns due to potential tumorigenicity, unwanted immune response inducement, and transmission of adventitious agents (Medvedev et al., 2010; Herberts et al., 2011; Lukomska et al., 2019). Another axis of development is on whole tooth regeneration. The healing potential of the tooth organogenesis technique is impressing, and more developments may include the use of 3D printing, and perhaps of iPSCs cells for the regeneration of whole teeth. When a body part can be regenerated or replaced (e.g., nose, ear), it is a considerable success (Zhou et al., 2018). Therefore, the technical opportunity to fully regenerate teeth should be fully exploited, and further developments should be explored to apply the technology to the clinic. Moreover, 3D bioprinting technology needs further development to improve the resolution, printing speed and biocompatibility, as well as to scale up the technology. In bioprinting, the highest resolution is obtained for a continuous line up of cells, in which cells are printed individually at defined positions and are in contact with each other. Currently, only laser-assisted bioprinters have a high resolution (approximately 10 μm) depending on the bioink and the cell concentration. Increasing the speed of printing is also an important development that is beneficial for cell viability, complex structure fabrication, and cost. Furthermore, it would be good to scale up bioprinted tissue constructs for clinical applications. Interestingly, in recent developments, an emerging technique named volumetric bioprinting has overcome these limitations and significantly improved the printing velocity and size of constructs with high complexity, providing new opportunities (Bernal et al., 2019). Additionally, any technological developments in handheld bioprinters will be beneficial for dentistry.
